# Detection of Non-Amplified *Mycobacterium tuberculosis* Genomic DNA Using Piezoelectric DNA-Based Biosensors

**DOI:** 10.3390/s100301846

**Published:** 2010-03-09

**Authors:** Thongchai Kaewphinit, Somchai Santiwatanakul, Chamras Promptmas, Kosum Chansiri

**Affiliations:** 1 Department of Biochemistry, Faculty of Medicine, Srinakharinwirot University, Sukhumvit 23, Bangkok 10110, Thailand; E-Mail: tkaewphinit@yahoo.com; 2 Department of Pathology, Faculty of Medicine, Srinakharinwirot University, Sukhumvit 23, Bangkok 10110, Thailand; E-Mail: titi41@yahoo.com; 3 Department of Clinical Chemistry, Faculty of Medical Technology, Mahidol University, Prannok, Bangkok, 10700, Thailand; E-Mail: mtcpm@mahidol.ac.th

**Keywords:** *Mycobacterium tuberculosis*, piezoelectric biosensor, non-amplified genomic DNA

## Abstract

Piezoelectric DNA-based biosensor technology was developed as a new method for detection of *M. tuberculosis*. This method consists of immobilizing a thiol-modified oligonucleotide probe on the gold electrode surface of a quartz crystal, using a self-assembled monolayer method. The advantage of this study is that a non-amplified genomic bacterial DNA target was used. Instead, the genomic DNA was digested by restriction enzyme to obtain DNA fragments containing the target sequence. The fabricated biosensor was evaluated through an examination of 200 samples. No cross hybridization were observed against *M. avium* complex and other microorganisms. This target DNA preparation, without PCR amplification, will reduce time, costs, and the tedious step of amplification.

## 1. Introduction

Tuberculosis (TB) is a disease caused by *Mycobacterium* spp. It is among the top ten causes of global mortality and morbidity, which had led to it becoming an important public health problem in Thailand. It is a slow-growing bacterium that needs 1–2 months for growing in culture [1,2]. The Ziehl-Neelsen (ZN) stain for direct specimen examination is a conventional diagnostic tools but lacks sensitivity [3,4]. Polymerase chain reaction (PCR) [5,6] is sensitive for detection of mycobacteria by using specific primers, but this amplification process requires additional processing time, reagents and devices, which affect the cost of assay. Moreover, PCR analysis needs well-trained personnel [7,8].

There has been increasing interest in biosensor technology for rapid and sensitive detection, especially the piezoelectric biosensor. This biosensor has the advantages that the detection method is free from radioactive or fluorescent tags [7,9,10]. In addition, this technique has potential to provide a qualitative and quantitative analysis. It is highly sensitive to mass on the surface of a quartz crystal with the high specificity of a bioreaction [9,11–13]. A thin oscillating gold quartz crystal surface generates intrinsic resonance frequency by mass attached, adhered or deposited onto the piezoelectric active surface.

DNA-based biosensor technology, which uses oligonucleotide hybridization detection, but does not require labeling, makes it attractive due to its ease-of-use. The piezoelectric quartz crystal is one of the candidate biosensor technology devices for detection of DNA hybridization. Piezoelectric DNA-based biosensor measures a frequency change between the frequency of the oligonucleotide probe immobilized on the quartz crystal and the frequency after the hybridization of DNA target [14]. There are many reports about the development of specific piezoelectric DNA-based biosensors for detection of many pathogenic bacteria such as *Escherichia coli* [15], *Aeromonas* spp. [7], and *Pseudomonas aeruginosa* [16]. The drawback of these studies is the applications of the methods they describe are only on PCR-amplified DNA.

In this study, piezoelectric DNA-based biosensor was developed as a new method for detection of *M. tuberculosis*. This method consists of immobilizing a thiol-modified oligonucleotide probe on a quartz crystal by the self-assembled monolayer method.

This biosensor was used for detecting target DNA by measuring the frequency change. The oscillation counting device was used for measuring the resonant frequency of the quartz crystal in all experiments in this study. The advantage of this study is that it uses a non-amplified genomic bacterial DNA target. Such target DNA preparation without amplification will reduce the time consuming step of amplification and costs. Finally, this study can be extended to develop new methods that are highly sensitive, specific, cheap, easy to use, and rapid for detection of *M. tuberculosis* in many fields of work such as clinical diagnosis, epidemiology study, and bioterrorist weapon survey.

## 2. Results and Discussion

### 2.1. Investigation of the Optimum Concentration of the DNA Probe for the Performance of the Sensor

**Figure 1.**
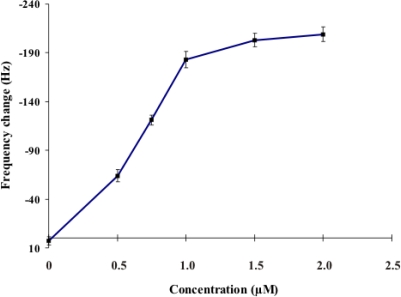
Resonant frequency changes of quartz crystal by thiol-modified oligonucleotide probe immobilization. Frequency changes were represented by the frequency differences between the frequency value of thiol-modified oligonucleotide probe immobilized on gold electrode (F1) and frequency value of original quartz crystal (F0) before immobilization (ΔF = F0-F1). Each data point represents the mean ± SD (n = 3).

Various concentrations of the 5′-thiolated probe (0, 0.5, 0.75, 1.0, 1.5, and 2.0 μM) were immobilized on the quartz crystal via gold-thiol-modified (SH-(CH_2_)_6_) reaction for 20 minutes at room temperature. After air-drying, the frequency change at each concentration of DNA probe immobilized (n = 3) was presented as mean ± S.D. as shown in Figure 1. The decrease of resonance frequency was almost linear with the increase in concentration from 0.5 μM to 1.0 μM of thiol-modified oligonucleotide probe, and was stable for concentration 1.5 and 2.0 μM.

Therefore, the concentration of thiol-modified oligonucleotide probe at 1.5 μM was chosen for use in all experiments of this study. At this concentration, the probe gave the highest frequency change of 1.5 μM synthetic DNA target hybridization. This result was different to to previous studies [17] that used the thiol-modified oligonucleotide probe at a concentration of 1.0 μM in piezoelectric biosensor. This concentration showed the minimal amount of probe to use in this system. This can lead to increased specificity due to the more stringent hybridization conditions.

### 2.2. Investigation of the Optimum Concentration of DNA target for Hybridization

**Figure 2.**
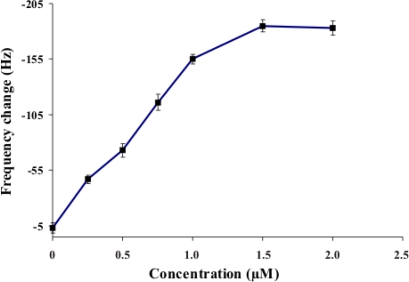
Resonant frequency change of quartz crystal by thiol-modified oligonucleotide probe for synthetic DNA target hybridization. The resonance frequency was measured and calculated for the frequency change. The frequency changes were represented by the frequency differences between this final value (F2) and the DNA probe immobilization (F1) as the baseline before the hybridization reaction (ΔF = F1-F2). Each data point represents the mean ± SD (n = 3).

Various concentrations of the synthetic DNA target (0, 0.25, 0.5, 0.75, 1.0, 1.5, and 2.0 μM) were hybridized with the 1.5 μM probe for 20 minutes at room temperature. After overnight air drying, the frequency change of each concentration of synthetic DNA target (n = 3) was presented as mean ± S.D. as shown in Figures 2. The optimum concentration of DNA probe can hybridize with the synthetic DNA target at the lowest concentration 0.25 μM. The frequency changes proportionally related to the increase of synthetic DNA target concentration: the decreasing resonance frequency was almost linear with the increase of synthetic DNA target concentration up to concentration of 1.5 μM.

At concentrations more than 1.5 μM, the frequency changes were stable. This indicated that the saturation concentration of synthetic DNA target hybridization was 1.5 μM. This result differs from that reported by Wu and colleagues [17] where the saturation of synthetic target DNA hybridization of others studies was 1.0 μM. Therefore, this system can detect the minimal synthetic target at 0.25 μM. This can improve the minimum detection limit of the proposed assay.

### 2.3. Improvement in the Hybridization of Bacterial Target DNA

**Figure 3.**
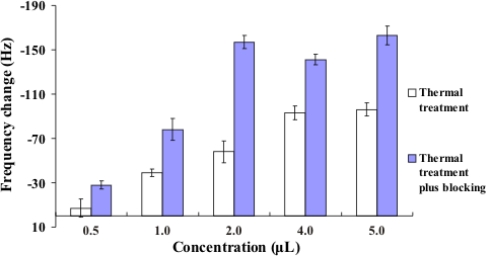
Resonant frequency change of DNA solution. *Btg*I (*Bst*DSI)-digested IS*6110* of *M. tuberculosis* genomic DNA was diluted with hybridization buffer before hybridization assay on piezoelectric biosensor. Each data point represents the mean ± SD (n = 3).

*M. tuberculosis* strain H37RVKK11-20 was chosen for studying DNA denaturation and the effect of the blocking oligonucleotides. All samples were hybridized with 1.5 μM of thiol-modified oligonucleotide probe for 20 minutes at room temperature. After air drying, the frequency change for each denaturation method (n = 3) was presented as mean ± S.D. as shown in Figure 3. The frequency change was reported as the difference between two stable frequency values based on Sauerbrey equation [18]. The frequency change of the DNA after thermal denaturation was compared to that after the annealing of the blocking oligonucleotides. The latter was higher than the former, which is in agreement with the reports of others [8,19]. This blocking can prevent the reannealing of single-stranded DNA to improve the efficiency of DNA target hybridization.

### 2.4. Evaluation of Piezoelectric DNA-Based Biosensor System

**Figure 4.**
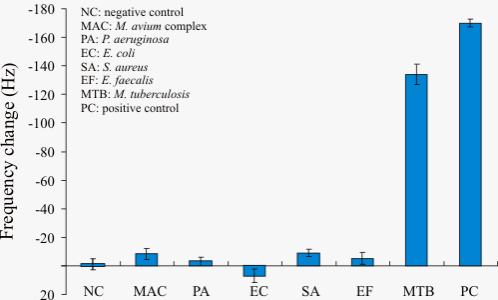
Resonance frequency change of quartz crystal for evaluation of piezoelectric DNA-based biosensor system. Six digested genomic DNAs were denaturated by thermal treatment plus treatment with blocking oligonucleotides. The hybridization buffer was used as negative control (NC). The synthetic DNA target at 1.5 μM of concentration was used as positive control (PC). Each data point represents the mean ± SD (n = 3).

The specificity was tested by using *Mycobacterium tuberculosis* H37RVKK11-20, *Mycobacterium avium complex*, *Escherichia coli*, *Pseudomonas aeruginosa*, *Staphylococcus aureus* and *Enterococcus faecalis*. The synthetic DNA target at concentration 1.5 μM was chosen as the positive control (PC) and the hybridization buffer as the negative control (NC). All samples were denatured by thermal denaturation plus blocking oligonucleotides and hybridized with 1.5 μM of thiol-modified oligonucleotide probe for 20 minutes at room temperature. After air-drying, the frequency change of each sample of denaturation methods (n = 3) was presented as mean ± S.D. as shown in Figure 4. The NC gave the lowest frequency change. *M. tuberculosis* gave higher frequency changes than those of *M. avium* complex, *E. coli*, *P. aeruginosa*, *S. aureus* and *E. faecalis*. The PC gave the highest frequency change. Therefore, the piezoelectric biosensor can differentiate *M. tuberculosis* from *M. avium complex* and other microorganisms after the thermal denaturation of DNA and the use of blocking oligonucleotides.

This result was similar to that reported in a previous study [5,6,20] that used IS*6110* target for detection of *M. tuberculosis* DNA amplified by PCR. The specific IS*6110* target was used to differentiate *M. tuberculosis* from non-*M. tuberculosis*.

**Figure 5.**
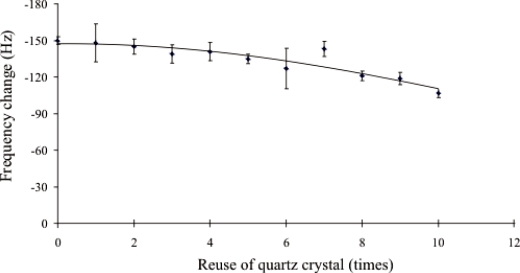
Resonance frequency change during testing of piezoelectric DNA-based biosensor reusability. 1.5 μM of synthetic DNA target was applied on the sensor at room temperature for 20 minutes to hybridize with probe. Each used sensor was added with 10 μL of 1mM HCl for one minute to wash out prior hybridized DNA target. The next cycle of hybridization was performed to test the capability of sensor. Each data point represents the mean ± SD (n = 3).

The reusability of the DNA-based biosensor system was evaluated by washing the used biosensor with 1 mM HCl solution for one minute to remove the attached target DNA. New DNA sample solution was then applied to the regenerated surface to test the hybridization ability of probe on the sensor surface. It was found that the washed sensor maintained its ability to hybridize with a new DNA sample at least 10 times without significant functional surface deterioration as shown in Figure 5.

### 2.5. Bacterial DNA Samples Analysis and Comparison

The *Bst*DSI-digested *IS6110* of genomic DNA from 200 samples (150 Ziehl-Neelsen (ZN)-positive samples and 50 ZN-negative samples) was tested by using the piezoelectric biosensor in comparison to the result of PCR technique (Table 1). The PCR products (using primers FMTB and RMTB) were detected using gel electrophoresis to confirm successful amplification of a 209 base pair PCR products (data not shown). One hundred and fifty ZN-positive samples had decreased frequency value (Hz) after the hybridization reaction with the DNA probe. The interaction with 50 ZN-negative samples did not result in a significant measurable frequency change and also no PCR amplicon band was observed. The data revealed that the results obtained from the piezoelectric biosensor correspond to those of PCR techniques. It can be concluded that specific IS*6110* gene sensor with DNA probe has high specificity for detection. For the detection in clinical samples, the proposed method presented 100% agreement to results using PCR, in regard to both to positive and negative samples. However, it had been reported that IS*6110* and its homolog can be detected by nested-PCR in some mycobacteriae other than *M. tuberculosis* [21]. It means that the specificity of these detection methods based on IS*6110* is not only on account of IS*6110* itself but also the possession of IS*6110* among bacteria strains. Therefore, this developed method has possibility to obtain false-positive results in clinical specimens containing non-*M. tuberculosis*.

Only some strains of *M. tuberculosis* possesses low copy numbers of IS*6110* as previous reported [22]. However, most of *M. tuberculosis* contains 2–5 copies. Practically, the digested DNA from sputum sample was diluted for 1:10 before application to the sensor. A 1:2 dilution of sample should be appropriate for detection of those possible low copy numbers of IS*6110* strains.

The normal clinical sample for diagnosis of Tuberculosis is lymph node aspirates, cerebrospinal fluid, ascitic fluid, pleural fluid, sputum, and others. The sputum specimen has the highest possibility to have heavy contamination with other organisms. The result showed that in the case of sputum samples, the performance of this biosensor system still remained satisfactory.

This piezoelectric DNA-based biosensor required three days for preparation of the DNA probe immobilized on the quartz crystal. In other report, Wu and colleagues [17] studied the circulating-flow system of piezoelectric biosensor for real-time detection of *E. coli* O157:H7 using one day for preparation. The disadvantage of this study is several steps of washing and drying. The development of this piezoelectric DNA-based biosensor to a real-time system is necessary in future studies. The advantage of this biosensor for detection of *M. tuberculosis* is no probe-labeling, ease-of-use, and direct detection of non-amplified genomic DNA, which eliminates the PCR step. This eliminates the requirement of well-trained personnel to handle the PCR technique, the additional processing time of the amplification process, and the reagents and device, which add to the cost of the assay.

## 3. Experimental Section

### 3.1. Reagents and Oligonucleotides

All oligonucleotides and the *Bst*DSI (*Btg*I) restriction enzyme used in this study were synthesized by Bio basic (Canada) and Sib enzyme (USA), respectively, listed in Table 2. The primers were newly designed to get PCR product size of 209 bp which is comparable to the size of DNA containing IS*6110* when cut by BtgI (218 bp). The PCR result using the new primers was comparable to previous reported primers [5,6,s^20^].

The forward and reverse primers for PCR in this study were designed based on the sequence of the IS*6110* gene retrieved from NCBI.

The probe used had the same sequence of the reverse primer used in this study. The primer sequence for PCR was utilized as a probe for this sensor. It was modified by adding a linker of TTTTTT to the 5′ end with the thiol-modified (SH-(CH_2_)_6_). The blockings (Blocking 1 and Blocking 2) were used for annealing with the ssDNA of the bacterial target sequence after denatuaration method.

4-(2-hydroxyethyl)-1-piperazine ethanesulfonic acid (HEPES), 30% hydrogen peroxide, 98% sulfuric acid, sodium chloride, hydrogen chloride, sodium hydroxide and 6-mercaptohexanol were purchased from Sigma Aldrich (USA). PCR reagents were purchased from Invitrogen (USA) and DNAzol^®^ was purchased from Invitrogen (USA) for isolation of bacterial genomic DNA. Other chemicals used were at analytical reagent grade, and distilled water (18.2 MΩ) was used throughout.

### 3.2. Apparatus

The 12 MHz AT-cut piezoelectric quartz wafer with gold electrode was used for preparing piezoelectric DNA-based biosensor. The gold electrode fabricated on the quartz wafer has diameter of 4 mm and thickness of 1000 Angstroms (Kyocera-Kinseki Company, Thailand). The model piezoelectric biosensor device (self-made) was used for all measurement in this study. An oscillation counting device was used for measuring the frequency change of the quartz crystal after the addition of immobilization material. The major components of this device are AVR-microcontroller, oscillation circuit and read out display. The frequency shifts were reported as the difference between two stable frequency values (±1 Hz) based on Sauerbrey equation [19].

### 3.3. Samples

Standard strains from cultivation including *M. tuberculosis* was provided from Department of Communicable Disease, Ministry of Public Health Thailand. *M. avium* complex, *E. coli*, *P. aeruginosa*, *S. aureus* and *E. faecalis* were provided from Department of Pathology, Faculty of Medicine, Srinakharinwirot University. The 200 clinical sputum samples; 150 samples as positive infection of *M. tuberculosis* and 50 samples as negative of non-*M. tuberculosis* and other bacteria were obtained from Department of Pathology, Faculty of Medicine, Srinakharinwirot University and Bureau of Tuberculosis, Ministry of Public Health Thailand.

### 3.4. Extraction, Fragmentation of Genomic DNA and PCR Amplification

Two loops full of standard strains cultured on medium and 0.5 mL of sputum samples were extracted in 1 mL DNAzol^®^ reagent by inverting the tube several times prior to centrifugation at 4,000 xg for 10 minutes. The DNA in supernatant was precipitated by adding 0.5 mL of cold absolute ethanol. The supernatant was discarded and the DNA pellet was washed twice with 1.0 mL of 70% ethanol by inverting the tubes 3 times. The mixture was then centrifuged at 13,000 xg for 5 minutes to allow DNA to settle and ethanol was removed by decanting. The genomic DNA was air-dried, distilled water was added and kept at 4 °C, and immediately used.

Genomic DNA of partial IS*6110* of purified *M. tuberculosis* DNA was performed by using *Bst*DSI (*Btg* I) restriction enzyme. All reactions were manipulated in 50 μL containing genomic DNA in 5 μL of 10X buffer, and 10 units of *Bst*DSI restriction enzyme. Sterile distilled water was added to adjust volume to 50 μL. The *Bst*DSI digestion was allowed to proceed at 37 °C for 14 hours. The reaction was inactivated by heating at 65 °C for 20 minutes and immediately used.

PCR amplification was performed using a DNA thermal cycler (MJ Research PTC-200 Peltier thermal cycler). The reaction was conducted in 25 μL volume containing 50 ng of genomic DNA in 10x PCR buffer (500 mM KCl, 200 mM Tris-HCl pH 8.4), 1 μM each of the primers (FMTB, RMTB), 100 μM each of dNTP, 1.5 mM MgCl_2_ and 1.5 U of *Taq* DNA polymerase. Amplification was performed with the following cycling conditions: 94 °C for 2 minutes; 30 cycles of 94 °C for 1 minute, 53 °C for 1 minute 72 °C for 1 minute and a final extension at 72 °C for 10 minutes. PCR amplicon was analyzed by electrophoresis on a 2.0% agarose gel.

### 3.5. Preparation of Piezoelectric DNA-Based Biosensor

The method used to prepare piezoelectric DNA sensor in this study was modified from Wu and colleagues [18]. Initially, the gold electrode surface was cleaned with hot Piranha solution consisting of H_2_O_2_ (30%) and H_2_SO_4_ in a 1:3 ratio for 30 seconds. The quartz crystals were then thoroughly washed with distilled water and used immediately afterward. The initial resonance frequency (F0) was measured as the baseline after overnight air drying. Then, 10 μL of the different concentration (0, 0.5, 0.75, 1.0, 1.5, and 2.0 μM) of thiol-modified oligonucleotide probe in the immobilization buffer (1M KH_2_PO_4_, pH 3.8) was immediately added and left to react on the surface of gold electrode to form self assembly monolayer (SAM) for 1 hour followed by immobilization buffer, distilled water rinsing. After washing step, the residual nonspecific binding on gold surface were blocked by 1 mM of 6-mercaptohexanol (MCH) for 1 hour. Then the quartz crystal was rinsed with distilled water air-dried, the resonant frequency (F1) was measured as the amount of added thiol-modified oligonucleotide probe on gold electrode surface. The quartz crystal at this stage was ready for the hybridization.

### 3.6. DNA Target Hybridization

The DNA target hybridization was performed by adding 10 μL of the each DNA target concentration (0, 0.25, 0.5, 0.75, 1.0, 1.5, and 2.0 μM) on to the surface of Au/thiol-modified oligonucleotide probe. The hybridization reaction was left for 20 minutes at room temperature. Each quartz crystal was washed well with hybridization buffer (150 mM NaCl, 20 mM Na_2_HPO_4_, and 0.1 mM EDTA, pH 7.4) to remove the unbound oligonucleotides followed by washing with distilled water, and air-drying. The new frequency (F2) was recorded. The frequency difference (ΔF) between resonance frequency of initial and final values was determined (ΔF = frequency of the immobilization probe on the quartz crystal (F1) - frequency of the hybridization reaction (F2), with F1 > F2. The frequency change (ΔF = F2 - F1) related to the amount of target DNA hybridized to the thiol-modified oligonucleotide probe immobilized on the quartz crystal surface.

### 3.7. Improvement of Hybridization of Bacterial Target DNA

**Figure 6.**
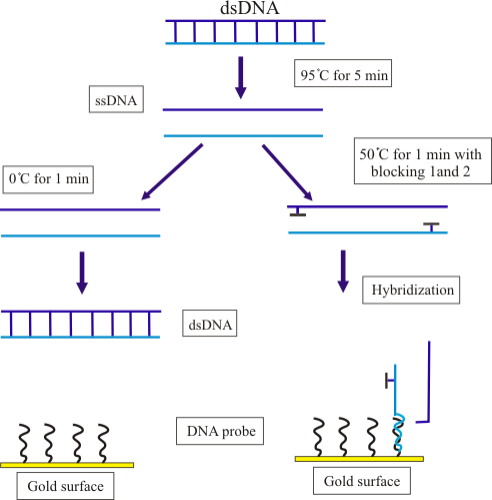
Schematic diagram of denaturation method. The bacteria dsDNA was denatured to ssDNA before hybridization with the DNA probe. Left: simple thermal denaturation, 95 °C for 5 minutes and on ice for 1 minute. Right: simple thermal denaturation plus blocking oligonucleotides, 95 °C for 5 minutes and cooled down at 50 °C for 1 minute to anneal the blocking oligonucleotides.

Generally, simple thermal treatment of bacterial target DNA is sufficient to give a significant analytical signal when amplified bacterial DNA fragment is used in DNA biosensor technique. But this treatment was not enough for non-amplified genomic DNA because of reannealing of bacterial target DNA. Minnuni and coworks [8,20] used the blocking oligonucleotides to block the bacterial target DNA after simple thermal treatment. This method increases the efficiency of hybridization between DNA probe and non-amplified genomic DNA. For this reason, different denaturation methods were studied as shown in Figure 6.

The optimal volume of the DNA targets loaded on the piezoelectric biosensor was determined. Aliquots of 0.5, 1, 2, 4, and 5 μL of digestion DNA target were diluted in hybridization buffer to a total volume of 10 μL before being added into the biosensor system.

### 3.8. Bacterial DNA Samples Analysis and Comparison

The piezoelectric DNA-based biosensor in this study for identification of *M. tuberculosis*, 150 ZN-positive and 50 ZN-negative from sputum samples were detected individually using the piezoelectric biosensor assay and PCR technique.

### 3.9. Statistical Analysis

Each experiment was performed in triplicate with different piezoelectric devices. All data were presented as the mean ± standard deviation (S.D.).

## 4. Conclusions

The piezoelectric DNA-based biosensor was developed for detection of *M. tuberculosis*. This method is promising to be a rapid and easy to use in diagnostic tests. This sensor can be tested with IS*6110* sequence also to differentiate *M. tuberculosis* from non-*M. tuberculosis*. This study will help the selection of a gene that is more suitable for detection of *M. tuberculosis*. For the sample detection, the concordance of the two methods with the samples incorporated in this study was complete. Moreover, piezoelectric DNA-based biosensor in liquid-flow system may be developed to further reduce the time and tedious steps of washing and drying the sensor preparation.

## Figures and Tables

**Table 1.** Samples identified with piezoelectric DNA-based biosensor, tested with specific sensors carrying the IS*6110* probes for genotyping, compared with the PCR method.

	**Number of samples**		
**Method**	**Positive**	**Negative**	**Total**
PCR technique	150	50	200
Piezoelectric biosensor	150	50	200

**Table 2.** The designated oligonucleotides for development of piezoelectric DNA-based biosensor.

**Name**	**Nucleotide sequence 5′ to 3′**
**Thiol-modified oligonucleotide probe**	SH-(CH_2_)_6_-TTTTTTGTGGCCATCGTGGAAGCGA
**Blocking 1**	CCTGCGAGCGTAGGCGTCGG
**Blocking 2**	ATCGTGGTCCTGCGGGCTTT
**Synthetic DNA target**	TCGCTTCCACGATGGCCAC
**Primer FMTB**	AAAGCCCGCAGGACCACGAT
**Primer RMTB**	GTGGCCATCGTGGAAGCGA
